# Mechanical Characteristics of 26H2MF and St12T Steels Under Torsion at Elevated Temperatures

**DOI:** 10.3390/ma18133204

**Published:** 2025-07-07

**Authors:** Waldemar Dudda

**Affiliations:** Faculty of Technical Sciences, University of Warmia and Mazury, Oczapowskiego 11, 10-719 Olsztyn, Poland; dudda@uwm.edu.pl

**Keywords:** torsional strength, yield stress, stress hypothesis, yield surfaces, heat-resistant steels

## Abstract

The concept of “material effort” appears in continuum mechanics wherever the response of a material to the currently existing state of loads and boundary conditions loses its previous, predictable character. However, within the material, which still descriptively remains a continuous medium, new physical structures appear and new previously unused physical features of the continuum are activated. The literature is dominated by a simplified way of thinking, which assumes that all these states can be characterized and described by one and the same measure of effort—for metals it is the Huber–Mises–Hencky equivalent stress. Quantitatively, perhaps 90% of the literature is dedicated to this equivalent stress. The remaining authors, as well as the author of this paper, assume that there is no single universal measure of effort that would “fit” all operating conditions of materials. Each state of the structure’s operation may have its own autonomous measure of effort, which expresses the degree of threat from a specific destruction mechanism. In the current energy sector, we are increasingly dealing with “low-cycle thermal fatigue states”. This is related to the fact that large, difficult-to-predict renewable energy sources have been added. Professional energy based on coal and gas units must perform many (even about 100 per year) starts and stops, and this applies not only to the hot state, but often also to the cold state. The question arises as to the allowable shortening of start and stop times that would not to lead to dangerous material effort, and whether there are necessary data and strength characteristics for heat-resistant steels that allow their effort to be determined not only in simple states, but also in complex stress states. Do these data allow for the description of the material’s yield surface? In a previous publication, the author presented the results of tension and compression tests at elevated temperatures for two heat-resistant steels: St12T and 26H2MF. The aim of the current work is to determine the properties and strength characteristics of these steels in a pure torsion test at elevated temperatures. This allows for the analysis of the strength of power turbine components operating primarily on torsion and for determining which of the two tested steels is more resistant to high temperatures. In addition, the properties determined in all three tests (tension, compression, torsion) will allow the determination of the yield surface of these steels at elevated temperatures. They are necessary for the strength analysis of turbine elements in start-up and shutdown cycles, in states changing from cold to hot and vice versa. A modified testing machine was used for pure torsion tests. It allowed for the determination of the sample’s torsion moment as a function of its torsion angle. The experiments were carried out at temperatures of 20 °C, 200 °C, 400 °C, 600 °C, and 800 °C for St12T steel and at temperatures of 20 °C, 200 °C, 400 °C, 550 °C, and 800 °C for 26H2MF steel. Characteristics were drawn up for each sample and compared on a common graph corresponding to the given steel. Based on the methods and relationships from the theory of strength, the yield stress and torsional strength were determined. The yield stress of St12T steel at 600 °C was 319.3 MPa and the torsional strength was 394.4 MPa. For 26H2MH steel at 550 °C, the yield stress was 311.4 and the torsional strength was 382.8 MPa. St12T steel was therefore more resistant to high temperatures than 26H2MF. The combined data from the tension, compression, and torsion tests allowed us to determine the asymmetry and plasticity coefficients, which allowed us to model the yield surface according to the Burzyński criterion as a function of temperature. The obtained results also allowed us to determine the parameters of the Drucker-Prager model and two of the three parameters of the Willam-Warnke and Menetrey-Willam models. The research results are a valuable contribution to the design and diagnostics of power turbine components.

## 1. Introduction

This paper refers largely to the mechanics of thermal stress, which is the basis for describing such phenomena as low-cycle thermal fatigue, thermo-mechanical destruction, thermo-plastic cracking, high-temperature cyclic creep, etc. These and other unrecognized phenomena occurring in structures made of modern heat-resistant steels are crucial for properly determining the safety of the device, its heating and cooling rate, service life and, in general, resistance to thermo-mechanical load cycles. The data presented in the paper allow for the improvement of strength hypotheses, especially in the context of heat-resistant materials. These materials show a significant disparity between the corresponding mechanical properties (yield stress or strength limits) in tension and compression, especially at elevated temperatures. Currently, the Huber–Mises–Hencky (HMH) hypothesis is most often used to determine the stress state, based on the assumption of the symmetry of critical parameters in tension and compression.

**Figure 1:**
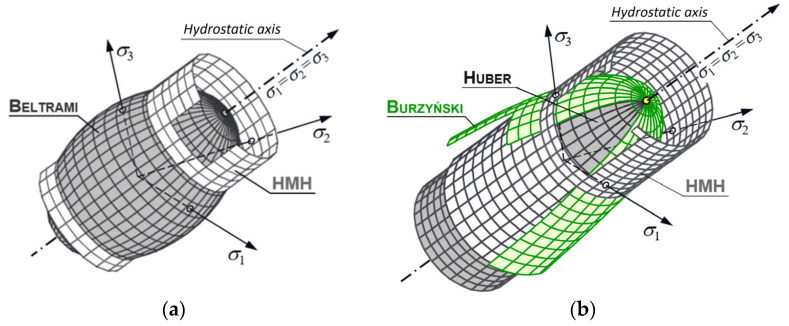
Drawings of boundary surfaces: Beltrami and HMH (**a**); Huber, Huber–Mises–Hencky (HMH), and Burzyński (**b**); $\sigma_{1}$, $\sigma_{2}$, $\sigma_{3}$—principal stresses.

In relation to materials not showing this symmetry of properties, the HMH hypothesis underestimates (in compression areas) and overestimates (in tension areas) the stress state. However, the original combined Huber hypothesis from 1904 [1] took into account this asymmetry, as it consisted in decomposing the strain energy Φ into the volumetric energy $\Phi_{v}$ and the distortional strain energy $\Phi_{f}$. However, in the description of the stress in the compressive stress area, only the shear energy is used, while in the case of the tension area, the sum of the shear and volumetric energy is used. In turn, the Beltrami stress measure from 1885 [2] always uses both energy components, and the von Mises hypothesis from 1913 [3] always uses only the distortional strain energy. During the First International Congress of Applied Mechanics in Delft (1924), Huber was persuaded by Mises and Hencky to reject Beltrami’s combined hypothesis in favor of an indefinitely cylindrical surface (only distortional strain energy), which he finally did in 1930. The original division of the energy hypotheses is presented in Table 1 and illustrated in Figure 1.

**Table 1:** The original division of energy hypotheses.

Author, Date of Hypothesis	Hypothesis Condition	Figure
Beltrami, 1885	Φ≤K always	Figure 1a
Huber, 1904	$\Phi_{v}+\Phi_{f}\;\;\leq K$ when $\sigma_{1}+\sigma_{1}+\sigma_{3}>0$ $\Phi_{f}\;\leq K$ when $\sigma_{1}+\sigma_{1}+\sigma_{3}<0$	Figure 1b
Mises, 1913 HMH, 1930	$\;\;\Phi_{f}\;\leq K$ always	Figure 1a,b
K—critical energy value determined experimentally

Many contemporary researchers emphasize the advantage of combined hypotheses [4,5,6,7,8], among them is the Burzyński hypothesis, which is an extension of the original combined hypothesis of Huber from 1904.

Burzyński formulates the equation of his hypothesis in such a way that it describes both the area of tensile and compressive stresses. This allows for taking into account materials showing differences between properties in tension and compression. In addition, he made his measure of effort dependent on properties in torsion. The construction of the hypothesis itself is such that with the same properties in tension and compression and with an appropriate value in torsion, it smoothly passes into the Huber–Mises–Hencky cylinder. The issue with this hypothesis and its use in relation to the numerical analysis of turbine blades has been widely described in works [9,10,11,12].

There are many equations describing anisotropic materials, such as the Drucker-Prager, Willam-Warnke, and Menetry–Willam models. However, they are most often used for materials such as concrete and other cohesive-friction materials (rocks, soil, and ceramics) [13,14,15,16].

The Burzyński model was corrected by Życzkowski in 1981 [17]. In 1999 Życzkowski, while analyzing discontinuous bifurcations of the Burzyński condition, demonstrated the parameters of this model through which we obtain, for example, the Beltrami-Schleicher ellipsoid, the Tor paraboloid, or the Drucker-Prager cone [18]. In all these cases we obtain circular sections in the deviatoric plane. In 2011 Pęcherski modifies the Burzyński hypothesis by introducing the third invariant of the stress tensor deviator, linking it with the Lode angle. This allowed modeling of non-circularity in the deviatoric section. Pęcherski et al. analyzed the range of variability of the modified model parameters and calibrated their values for the Inconel 718 alloy. They graphically presented the small depth of the plastic surface corrugation of this alloy both in the deviatoric cross-section and in the cross-section containing the hydrostatic axis [19].

A separate aspect in determining the strength of power turbine components is the estimation of the strength of components that are essentially subject to torsion. There is a lack of such studies in the literature, especially in the context of determining the yield stress and strength during torsion at elevated temperatures. For example, Khoddam and Hodgson conducted studies to determine the strain hardening indices during torsion using the Fields and Backofen method [20]. Zhang et al. studied the phenomenon of recrystallization and microstructure evolution in duplex stainless steel SAF2205 during torsional deformation at elevated temperatures [21], and Bhanuse and Jankar developed a double-twist torsion technique. They used 316 stainless steel as a sample material for experimental evaluation of recrystallization behavior and determination of the non-recrystallization temperature [22]. Hot torsion tests have been used to analyze and physically model the flow process and microstructure evolution of materials and alloys, e.g., API-X70 alloy steel [23], or to simulate thermo-mechanical processing of Al-Cu-Mg alloy [24]. There are works in which the behavior of Nb-Ti API 5L X80 alloy steel during plastic forming is assessed on the basis of torsion tests [25] or the parameters of hot plastic forming are determined for steels such as 30Cr25Ni32Mo3 steel [26] and X22CrNi17 steel [27]. Aiello et al. used hot torsion tests to induce cracks in 9Cr steel to assess the suitability of the material for nuclear reactor components [28]. Vuppala et al. presented an extended method for determining flow curves under shear loading using torsion tests. This method was used to determine strain rate constants for 16MnCrS5 steel [29]. Ballard et al. determined only the shear stress of a sample subjected to torsion as a function of temperature to understand the behavior of ordinary structural steel during fire conditions [30]. Hjorth investigated the effect of torsion at elevated temperatures on the change in the microstructure of AA6082 aluminum [31]. Zhou and Clode used the torsion test to improve the test data in the verification of the constitutive equations of AA5252 aluminum [32]. Li et al. proposed a high-temperature dynamic shear test method for Ti-1023 titanium alloy and investigated the microstructural evolution of the tested material at different temperatures [33].

Initial and extended research results aimed at determining the characteristics and material properties in tension and compression of these two steels were presented in [9,34,35].

The aim of the presented work is to determine the properties of heat-resistant steels St12T and 26H2MF, such as torsional yield stress and torsional strength, as well as strength characteristics in pure torsion and at elevated temperatures. This makes it possible to determine which of the two tested steels is more resistant to high temperatures. The results of the torsion test in combination with the results of tension and compression are intended to determine the yield surface of these steels. This is of fundamental importance in the design process and the analysis of the causes of turbine failures.

## 2. Tested Materials

Two of the most frequently dedicated steels for turbine components are ST12T and 26H2MF steels. These steels are similar in most alloying elements but contain different amounts of chromium (Table 2).

**Table 2:** Chemical composition of analyzed steels [34].

Steel		Chemical Composition (%)							
Grade	Signature	C	Si	V	Cr	Mn	Ni	Cu	Mo
26H2MF	24CrMoV55	0.23	0.52	0.25	1.54	0.30	0.12	0.17	0.60
St12T	X22CrMoV12-1	0.16	0.37	0.24	11.10	0.44	0.42	0.13	0.96

St12T steel belongs to the martensitic class of heat-resistant steels, with high corrosion resistance in steam medium while maintaining creep resistance at the same level as steels with lower chromium content. St12T steel is used for elements of rotors and blades of steam turbines operating at temperatures up to 600 °C and even 615 °C. On the other hand, 26H2MF steel is a ferritic steel resistant to creep, abrasion, and mechanical wear. It is used in the power equipment of turbines and boilers operating at temperatures up to 540 °C. It is most often used to make shafts, pin bolts, and nuts of boiler fittings [36,37].

Considering the scope of application, it was decided that static torsion tests will be carried out for the two heat-resistant steels at the temperatures listed in Table 3.

**Table 3:** Torsion test temperatures of analyzed steels.

Steel	Temperature [°C]				
St12T	20	200	400	600	800
26H2MF	20	200	400	550	800

## 3. Experimental Method

### 3.1. Sample Shape and Test Procedure

**Figure 2:**
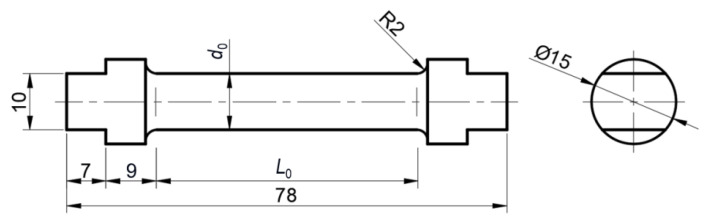
Shape and dimensions of torsion test samples at elevated temperatures.

To perform torsion tests, samples were prepared in the shape shown in Figure 2. Three samples were prepared from each of the tested materials for testing at a given temperature in the range of up to 600 °C (Table 3), and there was one sample for testing at 800 °C. Individual samples were numbered. The measuring lengths of all samples were $L_{0}=46$ mm and they were made with an accuracy of ±0.2 mm. The measuring diameters of each sample were measured with a micrometer and are included together with the testing temperatures in Table 4.

**Table 4:** Dimensions of St12T and 26H2MF steel samples.

St12T			26H2MF		
Sample No.	Diameter *d*_0_ [mm]	Temperature [°C]	Sample No.	Diameter *d*_0_ [mm]	Temperature [°C]
01	9.94	20	01	10.13	20
03	9.93		03	10.01	
04	10.05		04	10.02	
31	9.98	200	31	9.91	200
33	9.87		33	10.04	
34	9.95		34	9.92	
41	10.01	400	41	10.02	400
43	10.06		43	10.02	
44	10.00		44	10.16	
61	10.05	600	61	10.07	550
63	10.01		63	10.03	
64	9.88		64	10.04	
81	9.93	800	81	10.06	800

**Figure 3:**
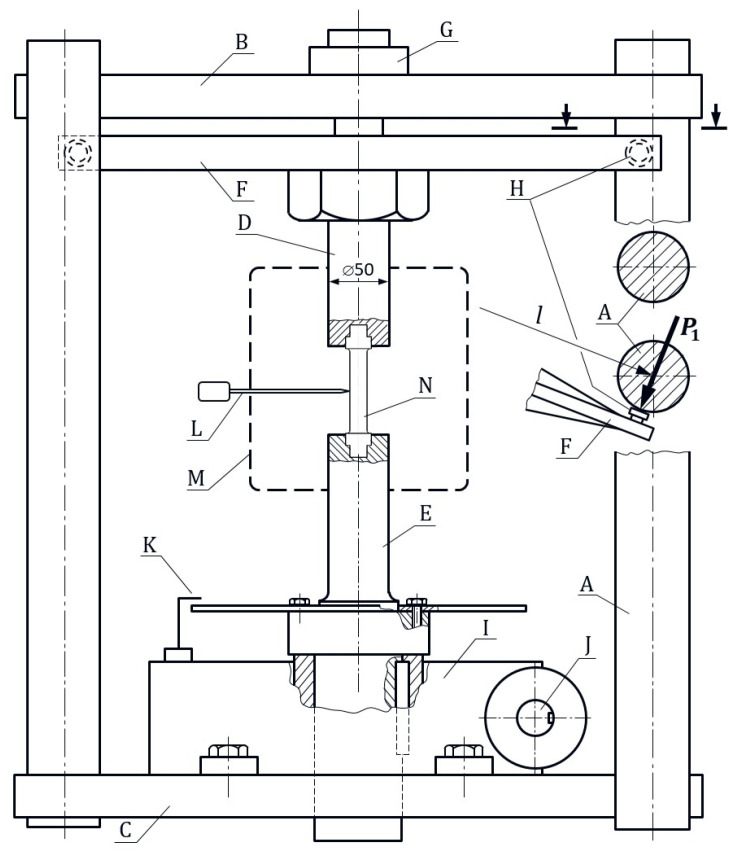
Schematic diagram of the stand for the torsion test of samples at elevated temperatures: machine columns (A), upper crossbeam (B), lower crossbeam (C), upper sample holder (D), lower sample holder (E), upper sample holder lever (F), lever bearing (G), strain gauges (H), worm gear (I), worm gear input shaft coupled with the main angle gauge (J), auxiliary angle gauge (K), thermocouple (L), heating chamber (M), sample (N).

The Heckert EUS-20 universal testing machine was adapted to perform the torsion test. The diagram of the test stand is shown in Figure 3. The screwed sample N is mounted in holders D and E. The sample holder D is mounted via bearings G in the upper crossbeam B of the machine. This allows the free rotation of the holder D together with its lever F around the vertical axis.

At the ends of lever F, symmetrically with respect to the axis of rotation, strain gauge sensors H are mounted, in contact with columns A of the machine. Simultaneous contact of these sensors with the columns is ensured by adjustment nuts. The signal from the strain gauges is sent via the L-Card E440 analog-to-digital converter to a computer with Power Graph 3.3.8 software for data collection and pre-processing. In order to convert the deformation indicated by the sensor into a force value, calibration is performed. It consisted in recording the readings of the strain sensor when it was loaded with forces of 40, 80, 120, 160, and 200 N, and then determining the average conversion factor value in this range. For this purpose, an AXIS FA200 force meter was used, with a range of 0 ÷ 200 N and an accuracy of 0.05 N. The coefficient determined as a result of this calibration allows the readings of the strain gauge to be converted into the values of forces $P_{1}$ and $P_{2}$ with an accuracy of 0.2 N. The torque moment of the sample is then calculated from the relationship:

(1) $$M_{s}=\frac{1}{2}\left(P_{1}+P_{2}\right)l,$$

where l is the distance between the points of contact of the gauges with the columns.

Using a keyway connection, the sample holder E was mounted in the socket of the worm gear, which was attached to the lower crossbeam C of the machine. The worm gear drive shaft was driven by an electric motor via a belt drive. The belt transmission ratio was selected so that the engine speed (adjusted by the inverter) could ensure the sample torsion in the range of 0.03–0.3 rad/min. The speed of the belt drive shaft was controlled by a tachometer. The worm gear drive causes the lower sample holder E to rotate. Then, with the upper holder D blocked by lever F, the sample is twisted. The auxiliary angle gauge disc K is attached to the holder E, while the main angle gauge J is attached to the input shaft of the gear. Twisting the sample by 1° requires the worm to rotate by 60°, which significantly improves the accuracy of the angle of rotation reading. After the sample is attached to the holders and the clearances are eliminated, the readings of the main angle gauge and dynamometers are reset to zero. We then closed the heating chamber M and heat the sample to the desired temperature. The accuracy of the temperature measurement with the thermocouple L was ±1 °C, and the accuracy of the temperature control was ±5 °C. After the sample is heated, the heater control module switches to the set temperature maintenance mode. A total of 10 min after reaching the set temperature, the torsion process was started at a speed of 0.03 rad/min, recording the torsion angle *φ* and the corresponding readings of the dynamometers $P_{1}$ and $P_{2}$. After the sample had clearly plasticized, the speed was increased to 0.3 rad/min. and torsion was continued until the sample was destroyed. After recording the data and calculating the torsion moment according to the relationship (1), a torsion diagram $M_{s}=f\left(\varphi \right)$ was prepared.

### 3.2. Method of Determining the Yield Stress and Torsional Strength

**Figure 4:**
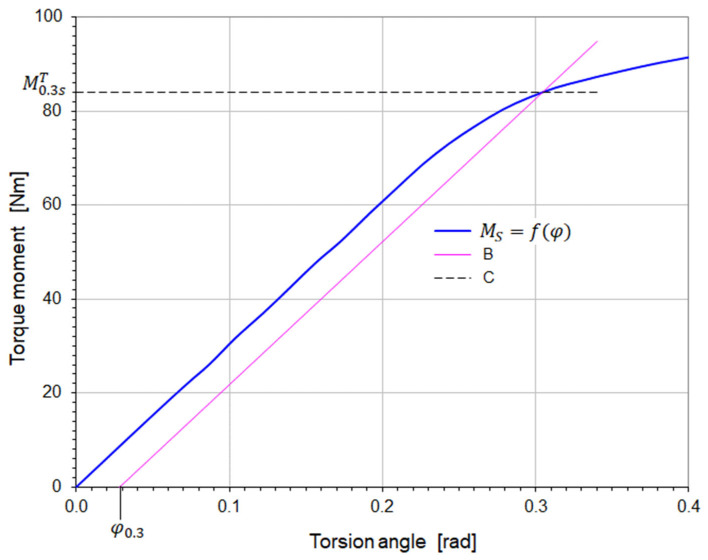
Graphical method for determining of the moment $M_{0.3s}^{T}$.

Using the enlarged fragment of the graph, the conventional yield strengths $R_{0.3s}^{T}$ are determined graphically. A straight line B is drawn on the graph (Figure 4), parallel to the proportional part of the torsion curve $f\left(\varphi \right)$, from the place corresponding to the conventional permanent shear strain $\gamma =\gamma_{0.3}=0.003$ [rad]. On the sample surface, the strain $\gamma_{0.3}$ corresponds to the torsion angle $\varphi_{0.3}$ such that

(2) $$\mathrm{tg}\gamma_{0.3}=r_{0}\;\varphi_{0.3}/L_{0}.$$

For small angles it is correct to assume $\mathrm{tg}\gamma_{0.3}=\gamma_{0.3}$, then

(3) $$\varphi_{0.3}=0.003L_{0}/r_{0}=0.006L_{0}/d_{0},$$

where $r_{0}$ and $d_{0}$ are the radius and diameter of the initial cross-section of the sample, and $L_{0}$ is the measuring length of the sample (Figure 2, Table 4).

Then, the straight line B is plotted for the torsion angle $\varphi_{0.3}$ given by the relationship (3). The ordinate C (Figure 4) of the intersection point of the straight line B with the torsion curve $M_{s}=f\left(\varphi \right)$ determines the moment $M_{0.3s}^{T}$, corresponding to the conventional yield strength in torsion. Finally, the value of the yield strength is calculated from the equation

(4) $$R_{0.3s}^{T}=M_{0.3s}^{T}/W_{0},$$

where $W_{0}$ is the cross-section index of the sample in torsion. In the case of samples with a circular cross-section, it is calculated from the formula

(5) $$W_{0}=\pi d_{0}^{3}/16.$$

The torsional strength of a material for clearly plastic materials, whose cross-section undergoes complete plasticization, is calculated from the relationship

(6) $$R_{s}^{T}=\frac{3}{4}M_{s}^{max}/W_{0},$$

where $M_{s}^{max}$ is the maximum torsional moment recorded during the torsion test of a given sample, until its failure.

## 4. Experimental Results

### 4.1. Torsion Diagrams for Heat-Resistant Steels St12T and 26H2MF

**Figure 5:**
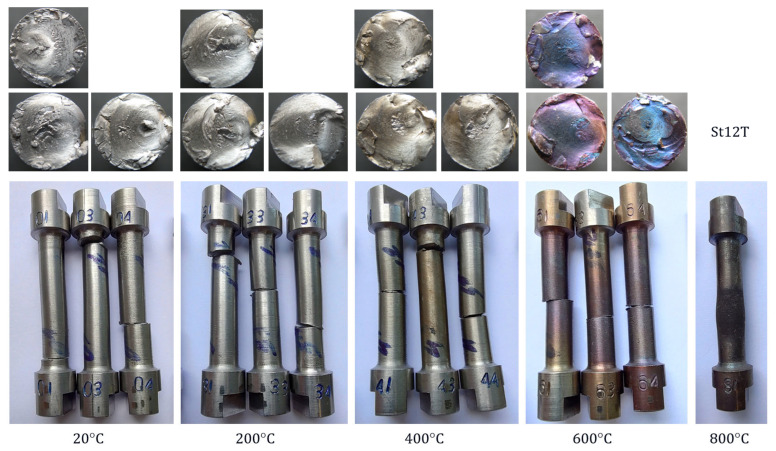
Samples of St12T steel after torsion tests.

**Figure 6:**
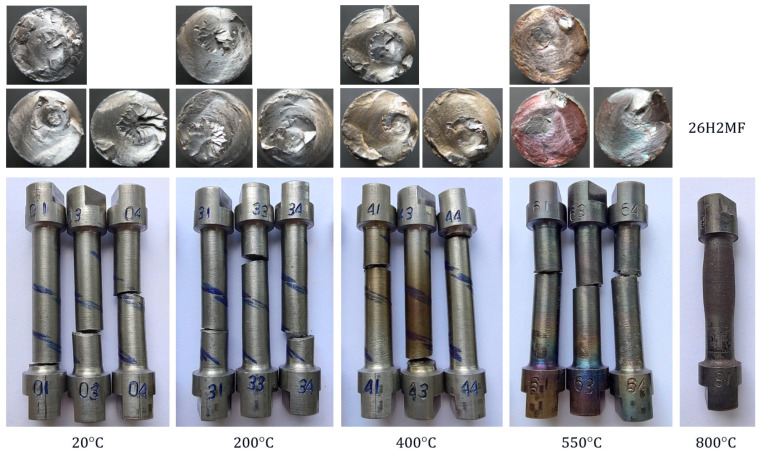
Samples of 26H2MF steel after torsion tests.

**Figure 7:**
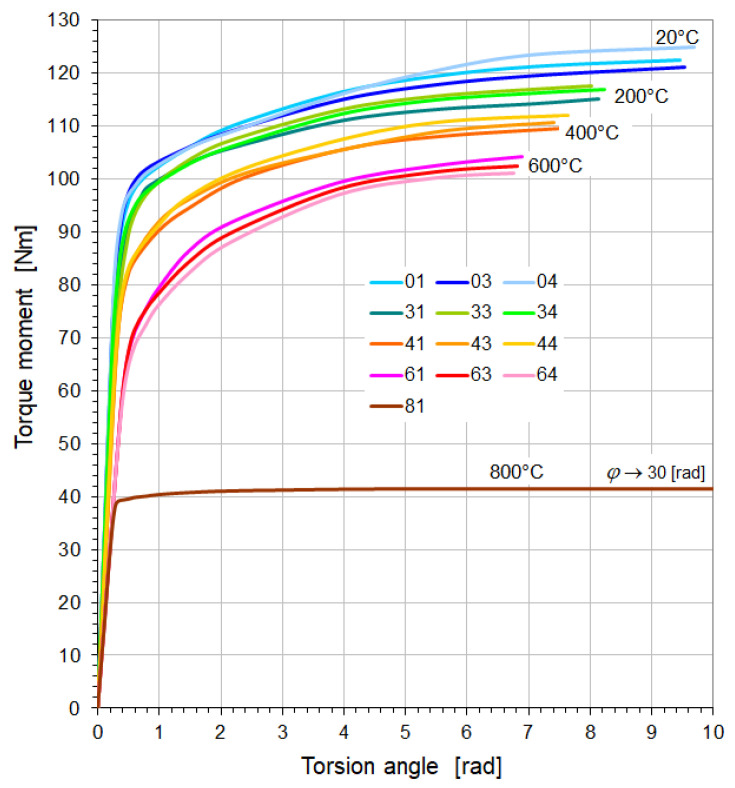
Torsion diagrams of St12T steel samples at elevated temperatures.

**Figure 8:**
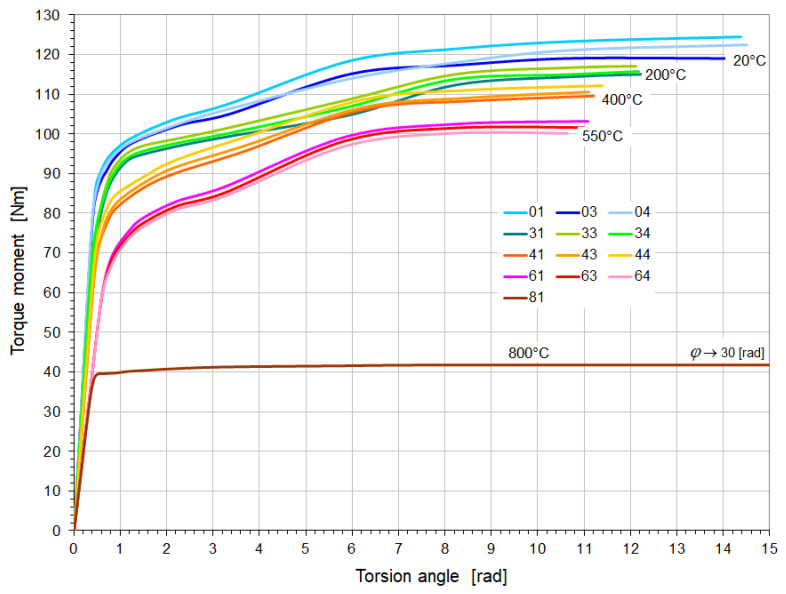
Torsion diagrams of 26H2MF steel samples at elevated temperatures.

Torsion tests were carried out on samples, the specifications and test temperatures of which are listed in Table 4. The fractures of individual samples together with the fracture planes are shown in Figure 5 and Figure 6. Their torsion diagrams $M_{s}=f\left(\varphi \right)$ at variable temperatures are shown in Figure 7 and Figure 8.

All samples and their failure planes shown in Figure 5 and Figure 6 show no visible defects; therefore, the tests can be considered valid.

The characteristics presented in Figure 7 and Figure 8 show that in the temperature range of 20–550 °C, 26H2MF steel shows approximately 50% larger plastic torsion angles before destruction compared to St12T steel. However, at torsion angles of approximately 4 [rad] before failure, the 26H2MF steel no longer showed any tendency to hardening, which is clearly visible especially in the case of samples tested at 550 °C. At 800 °C, both steels did not show hardening and became perfectly plastic in torsion. They were not destroyed, but their base (measuring) part swelled and shortened (Figure 5 and Figure 6). In both cases, the tests were stopped at a torsion angle of about 30 [rad] and with a practically unchanged torsional moment (Figure 7 and Figure 8).

### 4.2. Torsion Strength Properties of Heat-Resistant Steels St12T and 26H2MF

**Figure 9:**
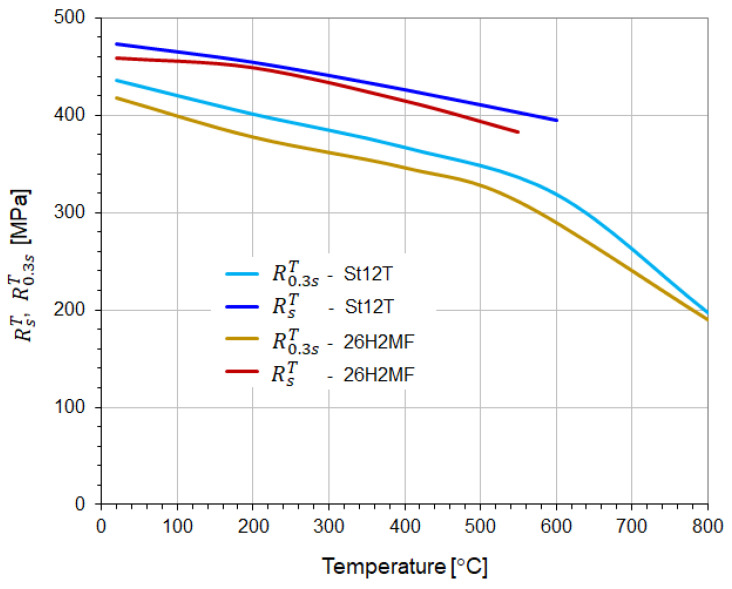
Average values of torsional strength $R_{s}^{T}$ and yield strength $R_{0.3s}^{T}$ at elevated temperatures in steel St12T and 26H2MF.

The values of torsional strength $R_{s}^{T}$ and yield stress $R_{0.3s}^{T}$ determined for individual samples, as well as their average values at a given temperature, are given in Table 5 and Table 6, respectively. The change in the average values of the determined properties of both heat-resistant steels with temperature is shown in Figure 9.

**Table 5:** Yield stress $R_{0.3s}^{T}$ and torsion strength $R_{s}^{T}$ of St12T steel.

Temperature [°C]	Sample No.	$R_{0.3s}^{T}$ [MPa]		$R_{s}^{T}$ [MPa]	
		Samples	Average Value	Samples	Average Value
20	01	435.6	436.4	476.4	473.0
	03	439.5		472.4	
	04	434.0		470.0	
200	31	404.8	401.8	442.7	454.1
	33	397.3		466.8	
	34	403.3		452.9	
400	41	368.1	367.5	412.4	425.9
	43	365.2		421.2	
	44	369.3		444.0	
600	61	321.1	319.3	392.1	394.4
	63	319.9		390.3	
	64	316.9		400.8	
800	81	197.7	197.7	-	-

**Table 6:** Yield stress $R_{0.3s}^{T}$ and torsion strength $R_{s}^{T}$ of 26H2MF steel.

Temperature [°C]	Sample No.	$R_{0.3s}^{T}$ [MPa]		$R_{s}^{T}$ [MPa]	
		Samples	Average Value	Samples	Average Value
20	01	421.4	417.6	457.5	458.5
	03	416.4		453.2	
	04	415.1		464.7	
200	31	376.8	377.5	451.3	448.7
	33	377.5		441.6	
	34	378.3		453.1	
400	41	342.3	346.1	416.1	414.8
	43	346.3		419.9	
	44	349.6		408.3	
550	61	314.2	311.4	386.0	382.8
	63	307.9		384.2	
	64	312.0		378.2	
800	81	190.1	190.1	-	-

The determined average value of the yield stress of St12T steel (Table 5) at a temperature of 20 °C was 436.4 MPa, and it decreased to 319.3 MPa at a temperature of 600 °C. In the case of 26H2MF steel, this limit (Table 6) was on average 417.6 MPa at 20 °C, and an increase in temperature to 550 °C caused a decrease in this limit to 311.4 MPa. In the case of both tested steels, a further increase in temperature to 80 °C caused a significant decrease in the yield stress value. In the case of St12T steel, this limit decreased to 197.7 MPa, and for 26H2MF steel to 190.1 MPa.

The temperature change from 20 °C to 600 °C for St12T steel and to 550 °C for 26H2MF steel (Table 5 and Table 6) caused a decrease in the torsional strength value from 473 MPa to 394.4 MPa (St12T) and from 458.5 MPa to 382.8 MPa (26H2MF), respectively. In the case of testing samples of both steels at temperatures of 800 °C, it was not possible to destroy the samples (Figure 5 and Figure 6), and at a torsion angle of 30 [rad] the tests were stopped. Hence, the torsional strength could not be formally determined at this temperature.

St12T steel is more resistant to temperature compared to 26H2MF steel. The values of its yield stress and torsional strength are higher than for 26H2MF steel at each of the temperatures: 20, 200, and 400 °C. Moreover, the values of both these properties are higher for St12T steel at 600 °C than for 26H2MF steel at 550 °C.

## 5. Using Torsion Test Results to Determine the Yield Surface

### 5.1. Burzynski’s Criterion

The results of the torsion test (Table 5 and Table 6) and the results of the tension and compression tests [9,12,34,35] were used to extend the Burzyński criterion to thermal stress. The critical stress parameters in his C5 hypothesis [38] for tension $k_{r}$, compression $k_{c}$, and torsion $k_{s}$ were related to the yield stress:

(7) $$k_{r}=R_{0.2r}^{T},\;k_{c}=R_{0.2c}^{T},\;k_{s}=R_{0.3s}^{T},$$

where $R_{0.2r}^{T}$ is the conventional yield stress determined in the uniaxial tension test, $R_{0.2c}^{T}$ is the conventional yield stress determined in the uniaxial compression test, and $R_{0.3s}^{T}$ is the conventional yield stress determined in the torsion test.

All three of these boundaries are defined at variable temperatures. Two equivalent parameters of the Burzyński hypothesis were introduced: the coefficient of asymmetry of the elastic region $\varkappa_{T}$ and the coefficient of plasticity $\nu_{T}$, in the form

(8) $$\varkappa_{T}=\frac{R_{0.2c}^{T}}{R_{0.2r}^{T}}\;,$$

(9) $$\nu_{T}=\frac{R_{0.2r}^{T}{\;R}_{0.2c}^{T}}{2{\left(R_{0.3s}^{T}\right)}^{2}}-1\;.$$

Then the hypothesis defining the surface of the onset of plasticity will take the form

(10) $$\sigma_{1}^{2}+\sigma_{2}^{2}+\sigma_{3}^{2}-2\nu_{T}\left(\sigma_{1}\sigma_{2}+\sigma_{2}\sigma_{3}+\sigma_{3}\sigma_{1}\right)+\;\left(\varkappa_{T}-1\right)R_{0.2r}^{T}\left(\sigma_{1}+\sigma_{2}+\sigma_{3}\right)-\varkappa_{T}{\left(R_{0.2r}^{T}\right)}^{2}=0.$$

In order to apply the above thermal stress hypothesis in practice for two heat-resistant steels (St12T and 26H2MF), the coefficients $\varkappa_{T}$ and $\nu_{T}$, corresponding to these steels, were determined. The values of these coefficients, calculated in accordance with Equations (8) and (9), are given in Table 7.

**Table 7:** Coefficients of plasticity and asymmetry at variable temperatures [9,12,34,35].

Steel	Temperature [°C]	Yield Strength [MPa]			Coefficient [-]	
		$R_{0.2r}^{T}$	$R_{0.2c}^{T}$	$R_{0.3s}^{T}$	$\varkappa_{T}$	$\nu_{T}$
St12T	20	720.3	786.0	436.37	1.091	0.4866
	200	656.7	731.7	401.76	1.114	0.4883
	400	608.0	666.3	367.51	1.108	0.4997
	600	487.0	639.3	319.28	1.313	0.5271
	800	160.0	290.0	197.65	1.813	1.1033
26H2MF	20	667.3	779.0	417.60	1.167	0.4905
	200	610.7	693.3	377.50	1.135	0.4855
	400	567.0	628.0	346.06	1.108	0.4866
	550	507.3	573.3	311.37	1.130	0.5001
	700	388.5	460.0	238.60 *	1.184	0.5696

* Value obtained from linear interpolation between temperatures 550 and 800 °C.

**Figure 10:**
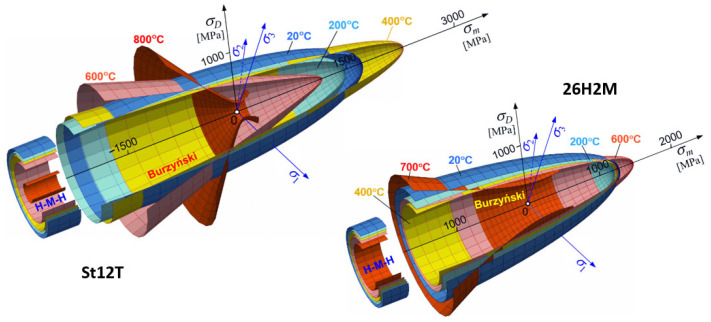
Evolution of the Burzynski and H-M-H yield surfaces of ST12T and 26H2MF steels with temperature.

The values in Table 7 allowed for the analysis of the influence of individual parameters on the geometry of the yield surface defined by the revised hypothesis (10). Figure 10 presents the evolution of the yield surface for the tested heat-resistant steels.

### 5.2. Drucker-Prager Model

The experimental data given in Table 7 can be used to determine the yield surface using the Drucker-Prager model [13], which can be written in terms of the principal stresses as

(11) $$\sqrt{\frac{1}{6}[{\left(\sigma_{1}-\sigma_{2}\right)}^{2}+{\left(\sigma_{2}-\sigma_{3}\right)}^{2}+{\left(\sigma_{3}-\sigma_{1}\right)}^{2}]}=A+B\;(\sigma_{1}+\sigma_{2}+\sigma_{3})$$

where parameters *A* and *B* are given by equations

(12) $$A=\frac{2}{\sqrt{3}}\left(\frac{R_{0.2r}^{T}{\;R}_{0.2c}^{T}}{R_{0.2r}^{T}+{\;R}_{0.2c}^{T}}\right)\;\;;\;\;\;B=\frac{1}{\sqrt{3}}\left(\frac{R_{0.2r}^{T}-{\;R}_{0.2c}^{T}}{R_{0.2r}^{T}+{\;R}_{0.2c}^{T}}\right)$$

Different uniaxial yield stresses in tension and in compression are predicted by the Drucker-Prager model. Their asymmetry coefficient is defined in the same way as in Burzyński’s case and can be expressed by the parameter B:

(13) $$\varkappa_{T}=\frac{R_{0.2c}^{T}}{R_{0.2r}^{T}}=\frac{1-\sqrt{3}B}{1+\sqrt{3}B}\;$$

However, this criterion was introduced to describe plastic deformations of soils. It and its many variants have been applied to rock, concrete, polymers, foams, and other pressure-dependent materials. Therefore, in order to assess the usefulness of this criterion for the strength analysis of heat-resistant steel elements, additional experiments should be performed in complex stress states.

### 5.3. Willam-Warnke and Menetrey-Willam Models

The Willam-Warnke and Menetrey-Willam yield criteria are functions that are used to predict when failure will occur in concrete and other cohesive-frictional materials such as rock, soil, ceramics, and fiber-reinforced concrete [14,15,16].

Before using the Willam-Warnke yield criterion to predict failure, the following must be determined: $\sigma_{t}$—the uniaxial tensile yield stress, $\sigma_{c}$—the uniaxial compressive yield stress, and $\sigma_{b}$—the equi-biaxial compressive yield stress.

In the case of the Menetrey-Willam model, the following parameters must be previously determined:$f_{t}$—the uniaxial tensile yield stress, $f_{c}$—the uniaxial compressive yield stress, and e—the parameter defining the roundness of the damage surface. The failure surface has sharp corners if e=0.5, and is fully circular around the hydrostatic axis if e=1 [15]. Alternatively, this model can be based on the parameters ${\bar{f}}_{t}$, ${\bar{f}}_{c}$, and ${\bar{f}}_{bc}$, which denote the actual uniaxial compressive strength, uniaxial tensile strength, and biaxial compressive strength, respectively [16].

The description of the yield surface of the tested heat-resistant steels using the Willam-Warnke and Menetrey-Willam models would require additional experiments, consisting of determining the yield stress under equi-biaxial compression at variable temperatures. Therefore, $\sigma_{b}$, $f_{b}$, or ${\bar{f}}_{bc}$ should be determined, while based on Table 7 we have $f_{t}={\bar{f}}_{t}=R_{0.2r}^{T}$ and $f_{c}={\bar{f}}_{c}=R_{0.2c}^{T}$. Then, in order to determine its applicability, the obtained model should be validated in a complex stress state.

## 6. Summary and Conclusions

The paper analyses the mechanical properties of two heat-resistant steels (St12T and 26H2MF) subjected to static torsion. Their yield and strength limits were determined when torsion occurred at different temperatures, and the data were presented in tables and graphs.

The conducted research is a response to the lack of experimental data on the behaviour of materials under torsion, which is important when designing power turbine components.

Particular emphasis was placed on comparing the results in the context of the asymmetry of mechanical properties in tension, compression, and torsion, which is crucial for validating the stress hypotheses. The research results were used to determine the parameters of the Burzyński hypothesis, extended by the effect of temperature, which enabled the presentation of the evolution of the yield surface geometry and further use of the results in numerical analyses and FEM simulations.

In addition, the use of the data contained in Table 7 to determine the parameters of the Drucker-Prager model is presented and the limitations in the use of these data in relation to the Willam-Warnke and Menetrey-Willam models are discussed. It was indicated that in this case it would be necessary to perform tests of samples under biaxial compression.

Practical conclusions from the pure torsion test of both tested heat-resistant steels:

1. The increase in temperature from 20 to 600 °C for St12T steel caused a decrease in the yield strength from 436.4 MPa to 319.3 MPa, while the torsional strength decreased from 473.0 MPa to 394.4 MPa.
2. The increase in temperature from 20 to 550 °C for 26H2MF steel caused a decrease in the yield strength from 417.6 MPa to 311.4 MPa, while the torsional strength decreased from 458.5 MPa to 382.8 MPa.
3. At 800 °C, St12T steel showed a yield strength of 197.7 MPa, while 26H2MF steel showed this limit at the level of 190.1 MPa.
4. Both steels tested at 800 °C were not destroyed despite the twisting of the samples to 30 radians, but they were shortened and swollen (Figure 5 and Figure 6), and after exceeding the yield point, the materials of these steels showed features of ideal plasticity in torsion $M_{s}=f\left(\varphi \right)=const.$ (no hardening—Figure 7 and Figure 8).
5. St12T steel has higher resistance to torsion at high temperatures than 26H2MF steel. Both its yield point of 394.4 MPa and torsional strength of 394.4 MPa at temperatures of 600 °C are higher than the yield point of 311.4 MPa and torsional strength of 382.8 MPa of 26H2MF steel determined at a temperature of 550 °C.

The research results presented in this paper offer new empirical data necessary in the modern design of energy machines.

## Data Availability

The original contributions presented in this study are included in the article. Further inquiries can be directed to the corresponding author.

## References

[1] Huber M.T. (2004). Specific work of strain as a measure of material effort. Arch. Mech..

[2] Beltrami E. (1885). Sulle condizioni di resistenza dei corpi elastici. Il Nuovo C..

[3] Mises R. (1913). Mechanik der festen Korper im Plastisch Deformablen Zustand. Nachr. Ges. Wiss. Gott..

[4] Altenbach H., Bolchoun A., Kolupaev V.A., Altenbach H., Öchsner A. (2014). Phenomenological Yield and Failure Criteria. Plasticity of Pressure-Sensitive Materials.

[5] Altenbach H., Eremeyev V.A., Lebedev L.P. (2010). On the existence of solution in the linear elasticity with surface stresses. ZAMM.

[6] Kolupaev V.A., Altenbach H., Bolchoun A. (2018). Comparison of strength criteria based on the measurements on concrete. J. Eng. Mech..

[7] Kolupaev V.A. (2018). Equivalent Stress Concept for Limit State Analysis.

[8] Altenbach H. (2010). Strength hypotheses—A never ending story. Czas. Tech. Politech. Krak..

[9] Dudda W., Ziółkowski P.J., Ziółkowski P., Bryk M., Badur J. (2024). A Concept of Thermal Effort for Heat-Induced Metal Plasticity. Materials.

[10] Ochrymiuk T., Dudda W., Froissart F., Badur J. (2021). Principles of Stress-Strength Modelling of the Highly Thermally Loaded Materials—The Influence of an Effect of Strength Differential on the Material Effort. Materials.

[11] Bryk M., Banaszkiewicz M., Kowalczyk T., Dudda W., Ziółkowski P. (2022). Slowly-closing valve behaviour during steam machine accelerated start-up. Case Stud. Therm. Eng..

[12] Dudda W., Kraszewski B. (2021). A theoretical validation of Burzyński hypothesis for a stress-strain analysis of heat-resistant steel. Case Stud. Therm. Eng..

[13] Drucker D.C., Prager W. (1952). Soil mechanics and plastic analysis for limit design. Q. Appl. Math..

[14] Willam K.J., Warnke E.P. (1975). Constitutive models for the triaxial behavior of concrete. Proc. Int. Assoc. Bridge Struct. Eng..

[15] Menetrey P., Willam K.J. (1995). Triaxial failure criterion for concrete and its generalization. ACI Struct. J..

[16] Dmitriev A., Novozhilov Y., Mikhalyuk D., Lalin V. (2020). Calibration and validation of the Menetrey-Willam constitutive model for concrete. Constr. Unique Build. Struct..

[17] Życzkowski M. (1981). Combined Loadings in the Theory of Plasticity.

[18] Życzkowski M. (1999). Discontinuous bifurcations in the case of the Burzyński-Torre yield condition. Acta Mech..

[19] Nowak M., Ostrowska–Maciejewska J., Pęcherski R.B., Szeptyński P. (2011). Yeld condition accounting for third invariant of stress tensor deviator. Eng. Trans..

[20] Khoddam S., Hodgson P. (2010). Post processing of the hot torsion test results using a multi-dimensional modelling approach. Mater. Des..

[21] Zhang J., Yao K., Chen H., Zhu G., Li F. (2018). Microstructure evolution of SAF2205 duplex stainless steel during torsion deformation at elevated temperature. Rare Met. Mater. Eng..

[22] Bhanuse M.M., Jankar P.D. (2013). Behavior of Steel under Elevated Temperature—Experimental work. Int. J. Adv. Sci. Tech. Res..

[23] Mirzakhani B., Salehi M.T., Arabi H., Khoddam S., Seyedein S.H., Aboutalebi M.R. (2008). Predication of temperaturę distribution in the hot torsion test specimen. Int. J. Eng. Sci..

[24] Carreño F., Cepeda-Jiménez C.M., Peñalba F., Carsí M., Ruano O.A. (2012). Simulation of hot rolling processing of an Al-Cu-Mg alloy by torsion tests. Mater. Sci. Forum.

[25] Martins F.H., Machado M.L. (2015). Estudo do comportamento termomecânico do aço API 5L X80 microligado ao Nb-Ti através de ensaios de torção a quente. Rev. Esc. Minas.

[26] Ebrahimi G.R., Keshmiri H., Arabshahi H. (2010). Mechanical Characteristics of Superaustenitic Stainless Steel Type 30Cr25Ni32Mo3 at Elevated Temperatures. Mater. Sci. Appl..

[27] Gusel L. (2019). Obtaining the stress-strain behaviour of stainless steel at elevated temperatures. Mater. Sci..

[28] Aiello G., Caes C., Lamagnere P., Martin A., Sauzay M. (2015). Tension–torsion ratcheting tests on 9Cr steel at high temperature. Nucl. Eng. Des..

[29] Vuppala A., Brüggemann H., Bailly D., Scharifi E. (2025). An Inverse Piecewise Flow Curve Determination Method for Torsion Tests at Elevated Temperature. Metals.

[30] Ballard T.J., Speer J.G., Findley K.O., De Moor E. (2021). Double twist torsion testing to determine the non recrystallization temperature. Sci. Rep..

[31] Hjorth A. (2018). The Effect of Deformation by Torsion and Subsequent Heat Treatment on the Microstructure of AA6082. Mater. Sci. Eng..

[32] Zhou M., Clode M.P. (1998). Thermal analysis of the torsion test under hot-working conditions. Comput. Mater. Sci..

[33] Li L., Wang Z., Ma W. (2022). Experimental Study on the High Temperature Impact Torsional Behavior of Ti-1023 Alloy. Materials.

[34] Dudda W. (2019). Influence of High Temperatures on the Mechanical Characteristics of 26H2MF and ST12T STEELS. Mater. Sci..

[35] Dudda W. (2020). Mechanical Characteristics of 26H2MF and St12T steels under compression at elevated temperatures. Strength Mater..

[36] Ennis P.J., Czyrska-Filemonowicz A. (2003). Recent advances in creep-resistant steels for power plant applications. Sadhana.

[37] Klotz U.E., Solenthaler C., Uggowitzer P.J. (2008). Martensitic-austenitic 9–12% Cr steels—Alloy design, microstructural stability, and mechanical properties. Mater. Sci. Eng. A.

[38] Burzyński W. (2009). Selected passages from Wodzimierz Burzyński´s doctoral dissertation: Study on material effort hypotheses. Eng. Trans..

